# A Hybrid Framework Integrating Past Decomposable Mixing and Inverted Transformer for GNSS-Based Landslide Displacement Prediction

**DOI:** 10.3390/s25196041

**Published:** 2025-10-01

**Authors:** Jinhua Wu, Chengdu Cao, Liang Fei, Xiangyang Han, Yuli Wang, Ting On Chan

**Affiliations:** 1 China Railway Siyuan Survey and Design Group Co., Ltd., Wuhan 430063, China; wujh_rs@outlook.com (J.W.); 003095@crfsdi.com (C.C.); 2 School of Geography and Planning, Sun Yat-sen University, Guangzhou 510006, China; hanxy35@mail2.sysu.edu.cn (X.H.); chantingon@mail.sysu.edu.cn (T.O.C.); 3 Department of Land Surveying and Geo-Informatics, The Hong Kong Polytechnic University, Hong Kong 999077, China; yuliwang2028@gmail.com

**Keywords:** GNSS displacement monitoring, time series decomposition, attention-based neural networks, multiscale time series modeling, landslide displacement prediction

## Abstract

Landslide displacement prediction is vital for geohazard early warning and infrastructure safety. To address the challenges of modeling nonstationary, nonlinear, and multiscale behaviors inherent in GNSS time series, this study proposes a hybrid predicting framework that integrates Past Decomposable Mixing with an inverted Transformer architecture (PDM-iTransformer). The PDM module decomposes the original sequence into multi-resolution trend and seasonal components, using structured bottom-up and top-down mixing strategies to enhance feature representation. The iTransformer then models each variable’s time series independently, applying cross-variable self-attention to capture latent dependencies and using feed-forward networks to extract local dynamic features. This design enables simultaneous modeling of long-term trends and short-term fluctuations. Experimental results on GNSS monitoring data demonstrate that the proposed method significantly outperforms traditional models, with R^2^ increased by 16.2–48.3% and RMSE and MAE reduced by up to 1.33 mm and 1.08 mm, respectively. These findings validate the framework’s effectiveness and robustness in predicting landslide displacement under complex terrain conditions.

## 1. Introduction

Landslides are widespread geohazards that can lead to severe damage to transportation infrastructure, urban development zones, and life safety across varied topographies [1,2,3]. The real-time monitoring and predicting of landslide displacement are critical not only for disaster prevention but also for enhancing infrastructure resilience in transportation, mining, and urban sectors [4]. Early detection of displacement anomalies enables timely mitigation measures and supports decision-making under uncertainty [5]. Accurately modeling landslide behavior remains challenging, as slope deformation processes are shaped by complex and interacting geological, hydrological, and environmental factors [6], often leading to highly nonlinear and dynamic displacement responses [7]. This complexity necessitates robust and adaptive predicting frameworks capable of capturing diverse deformation patterns and responding to external triggers [8,9].

Traditional displacement prediction approaches evolve from theory-driven models to data-driven strategies, including physical models, empirical-statistical methods, and time series analysis techniques [10]. Physical models grounded in soil mechanics, such as limit equilibrium or finite element methods, are often restricted by the difficulty of accurately capturing geotechnical parameters in field conditions [11,12]. Empirical-statistical methods construct predictive relationships between landslide displacement and external triggers such as precipitation, reservoir level, or seismic intensity based on historical correlations. Despite their applicability in data-scarce settings, these models typically exhibit limited capacity in capturing nonlinear system behavior and ensuring cross-site or temporal generalizability [13,14]. Time series analysis techniques, including linear regression, polynomial fitting, and ARIMA, have been used for landslide monitoring due to their simplicity and low computational cost [15,16]. However, they generally assume data stationarity and linearity, limiting their ability to cope with nonlinear, seasonal, or trend-shifting signals [17]. These limitations highlight the broader challenge of applying traditional models to landslide systems with complex temporal dynamics. For instance, Welsch and Heunecke highlighted that congruence and kinematic models, though effective under static or quasi-static conditions, are limited in capturing the temporal variability of displacement in dynamic slope environments [18]. This underscores the need for more flexible and data-driven approaches, such as deep learning-based time series models, which are better suited to handle nonlinear dependencies, latent patterns, and evolving external influences [19].

With the rise of data-driven technologies, machine learning (ML) and deep learning (DL) methods have been introduced to landslide prediction. Support vector machines (SVM) [20,21], extreme learning machines (ELM) [22,23], and random forests (RF) [24,25] have all demonstrated improved performance over classical models. Deep models like the long short-term memory (LSTM) [26], gated recurrent unit (GRU) [27], and Bidirectional LSTM (Bi-LSTM) [28] are capable of learning long-term dependencies and nonlinear features, which makes them well-suited for landslide displacement time series. These recurrent neural network-based (RNN) methods have shown strong predictive ability in GNSS deformation analysis [29], attributed to their temporal memory structure. However, their performance may degrade in the presence of complex trend-seasonal interactions or noise contamination, as RNNs often lack explicit mechanisms for multiscale pattern separation.

To overcome these limitations, some studies have introduced time series decomposition techniques to enhance neural network modeling by leveraging structured time–frequency components such as trends, seasonality, and residuals. Li et al. proposed a hybrid convolution neural network long short-term memory (CNN-LSTM) model combined with the improvement of complete ensemble empirical mode decomposition with adaptive noise (ICEEMDAN), approximate entropy (ApEn), achieving effective predictions under nonstationary conditions [30]. Wang et al. developed a Variational Mode Decomposition (VMD) with Sparrow Search Optimization (SSO) and LSTM (VMD–SSO–LSTM) model integrating signal decomposition and swarm intelligence optimization, significantly improving landslide displacement prediction accuracy [31]. Time series decomposition techniques facilitate the extraction of intrinsic temporal structures, thereby enhancing model interpretability and predictive accuracy [32].

To further enhance the capability of traditional time series prediction models in capturing temporal dependencies, attention-based neural network models have been adapted. Kuang et al. introduced an Attentive Graph Neural Network (AGNN) for landslide displacement predicting, outperforming traditional DL models [33]. Bai et al. developed a VMD and Dual-stage Attention-based Recurrent Neural Network (DA-RNN) landslide prediction model that combines signal decomposition and dual-stage attention mechanisms to improve displacement predicting accuracy by modeling trend, periodic, and random components separately [34]. Building on the success of attention mechanisms in recurrent architectures, more recent models have adopted Transformer-based designs to further improve scalability and representation capacity. Originally developed for natural language processing (NLP), Transformer architectures [35,36] use multi-head attention to handle long sequences and complex inter-variable dependencies. For instance, Ge et al. proposed a lightweight and interpretable Transformer-based network (LiteTransNet) model that leverages localized self-attention to enhance both prediction accuracy and temporal interpretability in landslide displacement predicting [37]. Wu et al. proposed a Cross-Attention Stacked Transformer model incorporating cross-attention to fuse exogenous and endogenous variables, which significantly outperformed classical baselines, achieving RMSE reductions of over 65% at typical monitoring sites [38]. Despite these advancements, existing Transformer-based models often lack mechanisms for multiscale temporal decomposition and component-wise modeling, limiting their robustness under non-stationary conditions [39,40].

To address the limitations of existing models in capturing non-stationary and multiscale displacement patterns, this study proposes a hybrid predicting framework that integrates the Past Decomposable Mixing (PDM) module with an inverted Transformer architecture (iTransformer). The PDM module enables multiresolution trend-seasonal decomposition, while iTransformer enhances temporal abstraction and cross-variable interaction modeling. Together, they form a unified approach tailored for GNSS-based landslide displacement predicting. The main contributions of this study are as follows:

(1) A novel PDM module is integrated into the framework to perform structured decomposition and information mixing across temporal scales, improving the interpretability and robustness of time series feature extraction.
(2) An inverted Transformer architecture is introduced to decouple temporal and variable dimensions in multivariate modeling, enhancing the ability to capture long-range dependencies and cross-variable dynamics.

To evaluate the proposed framework, a GNSS-monitored landslide case in Chifeng, Inner Mongolia, China, is selected, enabling in situ validation under complex geological and climatic conditions.

## 2. Study Area and Data

**Figure 1:**
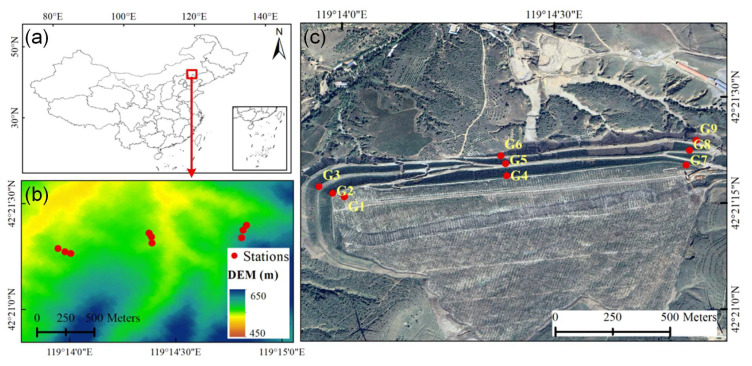
Study area: (**a**) Geographical location of the landslide in Chifeng City, Inner Mongolia Autonomous Region, China. (**b**) DEM. (**c**) Land cover around landslide.

In this study, a representative landslide located in Chifeng City, Inner Mongolia Autonomous Region, China, was selected to evaluate the predictive accuracy and performance of the proposed method under complex geological conditions, as shown in Figure 1. The landslide lies within the transitional zone between temperate arid and semi-arid regions, exhibiting distinct climatic characteristics. The annual mean temperature is approximately 5 °C, with an average annual precipitation of around 400 mm. Rainfall is highly seasonal, primarily concentrated between June and August during the summer monsoon, which also corresponds to the peak period of regional landslide activity. The landslide body extends linearly along the mountain slope in a strip-like pattern, with the overall slope surface developing in a stepped morphology and showing distinct multi-level sliding features. The stratigraphy is dominated by layered fractured rock masses, mainly consisting of interbedded sandstone and mudstone, and strongly controlled by structural planes. Due to prolonged weathering and gravitational loading, the mudstone layers are prone to softening and strength degradation. When infiltrated by rainfall or surface runoff, these weakened zones lead to reduced shear strength, making it easier for slip surfaces to form along the interfaces between sandstone and mudstone layers, thus triggering slope failures. The area is characterized by loose geological structures, high permeability, and steep slopes, representing a typical rainfall-induced landslide-prone environment, and thus provides a representative case for developing and validating GNSS-based displacement predicting models.

**Figure 2:**
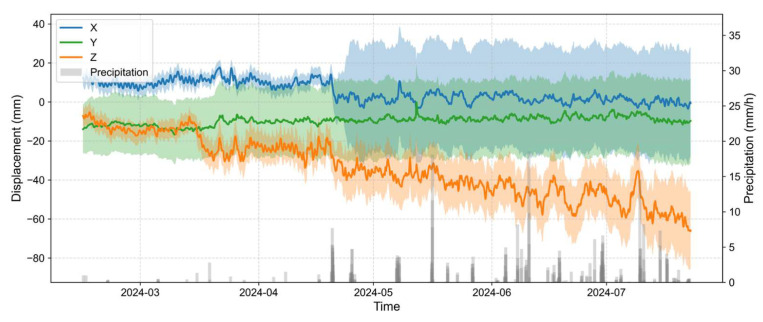
Time series of landslide displacements in the X (blue), Y (green), and Z (orange) directions from nine GNSS stations and precipitation intensity. Solid lines represent the average displacement across all stations in each direction, while shaded areas indicate the corresponding standard deviation.

To achieve continuous and high-precision monitoring of three-dimensional surface deformation, a spatial GNSS monitoring network was established, as illustrated in Figure 1. A total of nine GNSS observation stations (labeled G1 to G9) were evenly distributed across the slope surface in the X, Y, and Z directions. The stations continuously collected GNSS data from 15 February to 23 July 2024 (160 days). Each receiver recorded raw measurements, including carrier phase, pseudorange, and multipath indicators, using an integrated onboard data logging system. High-precision 3D coordinates were computed via static baseline differential processing, and epoch-differenced hourly displacement sequences were derived. To suppress colored noise in the GNSS displacement time series, an Unscented Kalman Filter (UKF) was applied, and the resulting mean displacements of all stations are presented in Figure 2. For the UKF model, the state vector included the true displacement, its rate of change, and a colored noise term. The process model assumed constant-velocity motion with a first-order Gauss–Markov process for colored noise, and the observation model treated measured displacement as the sum of the true state and measurement noise. Measurement noise covariance was set based on GNSS receiver precision under short-baseline conditions. The initial states were derived from the first hour of data, and the initial covariance was determined based on the variability observed during this early period. Filtering was performed strictly in a forward-only manner, using only past and current observations to avoid future information leakage and ensure suitability for predictive modeling and real-time landslide monitoring.

All monitoring equipment deployed in the field adopted the Unistrong MIS30 universal GNSS receiver, supplied by Guangzhou Geoelectron Technology Co., Ltd. (Guangzhou, China). This device supports multi-constellation and multi-frequency observations (including BDS, GPS, GLONASS, and Galileo), and is capable of high-precision static positioning. Under short-baseline conditions, the horizontal positioning accuracy reaches ±2.5 mm, and vertical accuracy is approximately ±5 mm, thus satisfying the millimeter-level precision requirements for landslide displacement monitoring.

In addition to displacement observations, meteorological data were collected using a local rain gauge installed near the landslide stations, which recorded hourly precipitation measurements over the same monitoring period. Considering the uneven temporal distribution of precipitation in the study area, cumulative precipitation features over multiple time windows (24 h, 48 h, 72 h, …, up to 7 days) were derived to better characterize the hydrological influence on landslide displacement. This multiscale representation enables the model to capture both short-term triggering events and longer-term soil moisture accumulation effects. Furthermore, a binary indicator (isRain) was incorporated as part of the cumulative precipitation feature set to enhance the model’s sensitivity to infrequent yet impactful precipitation occurrences.

## 3. Methodology

**Figure 3:**
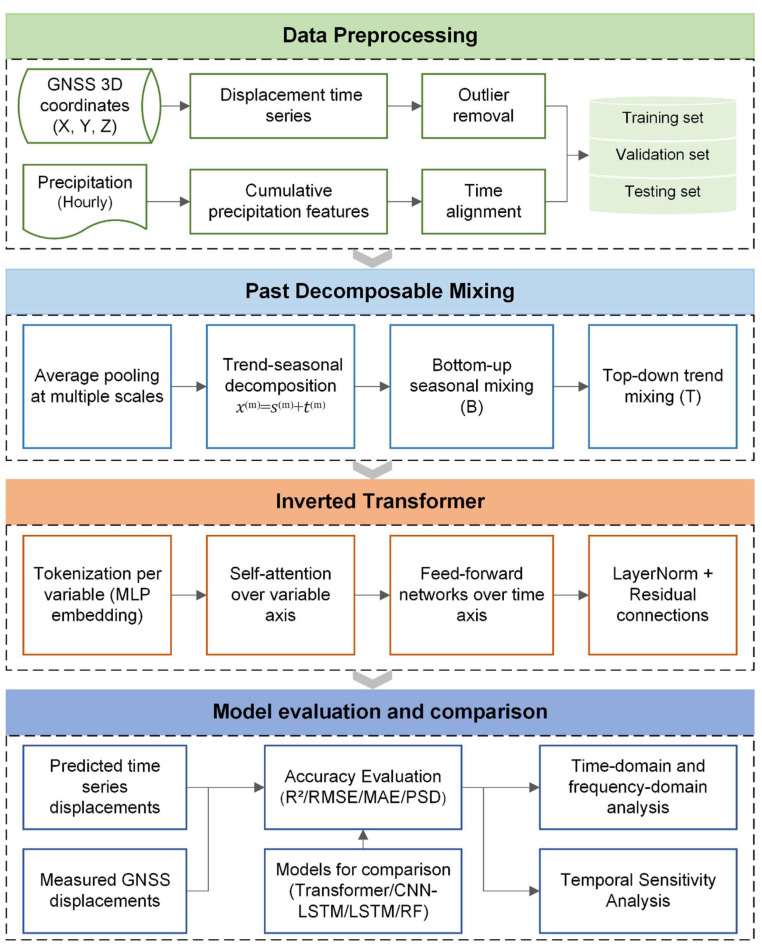
Flowchart of the proposed PDM-iTransformer framework.

To address the challenges of nonlinear and multi-scale modeling in landslide displacement processes, this study proposes a PDM-iTransformer-based prediction framework consisting of two core modules: past decomposable mixing and inverted Transformer modeling, as shown in Figure 3. Prior to model input, GNSS displacement and precipitation data undergo a preprocessing stage involving temporal alignment, feature construction, and dataset partitioning, which serves as foundational preparation but is not considered part of the proposed method itself. The PDM-iTransformer then applies multi-scale decomposition and trend–seasonal fusion to extract essential temporal structures, followed by an inverted Transformer architecture that models inter-variable dependencies and temporal dynamics to enable accurate landslide displacement predicting. Comparative experiments on landslide datasets demonstrate the model’s effectiveness and robustness under complex displacement conditions.

### 3.1. Past Decomposable Mixing (PDM) Module

**Figure 4:**
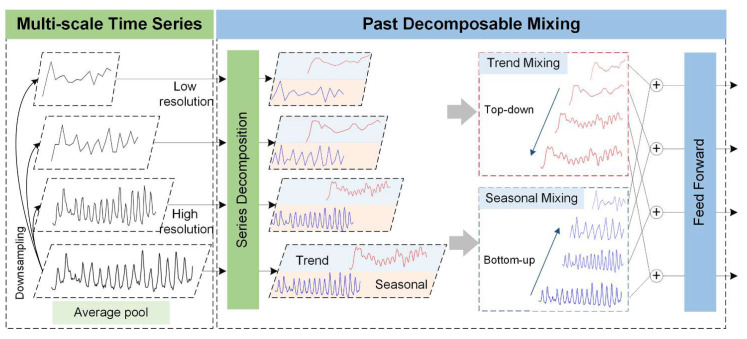
Structure of PDM.

To effectively model non-stationary and multiscale time series data, the Past Decomposable Mixing (PDM) module is introduced to automatically capture essential temporal features from past observations [41]. The core idea of PDM is to decompose input time series into multiple resolutions and perform structured mixing across scales, enabling a clear separation of complex temporal patterns and enhancing multiscale representation learning, as shown in Figure 4.

In practice, PDM first constructs multi-scale sub-sequences via temporal downsampling and then applies average pooling to extract both fine-grained and coarse-grained temporal dynamics. Each of these multiscale sequences is then decomposed into seasonal components and trend components, as formulated below:

(1) $$x^{\left(m\right)}=s^{\left(m\right)}+t^{\left(m\right)},\;\;\;\;\;\;m=0,1,\ldots ,M$$

where $x^{\left(m\right)}$ denotes the input sequence at scale *m*; *M* is the number of the scales; $s^{\left(m\right)}$ and $t^{\left(m\right)}$ represent the seasonal and trend components at that scale, respectively.

Recognizing the distinct temporal characteristics of these components, PDM adopts differentiated information mixing strategies: bottom-up aggregation is applied to seasonal components to pass fine-scale details upward, while top-down guidance is applied to trend components to propagate coarse-scale stability downward. These operations can be formalized as:

(2) $$s^{\left(m\right)}\leftarrow s^{\left(m\right)}+B(s^{\left(m-1\right)}),\;\;\;\;\;\;t^{\left(m\right)}\leftarrow t^{\left(m\right)}+T(s^{\left(m-1\right)})$$

where *B*(⋅) and *T*(⋅) denote the bottom-up seasonal mixing and top-down trend mixing layers, respectively, both implemented as two-layer feedforward networks with GELU activation along the temporal axis [41].

Compared to traditional single-scale or static decomposition methods, PDM offers a more adaptive and interpretable approach to handling real-world time series with intertwined seasonal fluctuations and long-term trends. When combined with Transformer or MLP-based predicting networks, the PDM architecture provides a unified framework with component-wise disentanglement, hierarchical fusion, and bidirectional cross-scale interaction, ultimately enhancing prediction accuracy and robustness across a wide range of time series predicting tasks [42].

### 3.2. iTransformer

**Figure 5:**
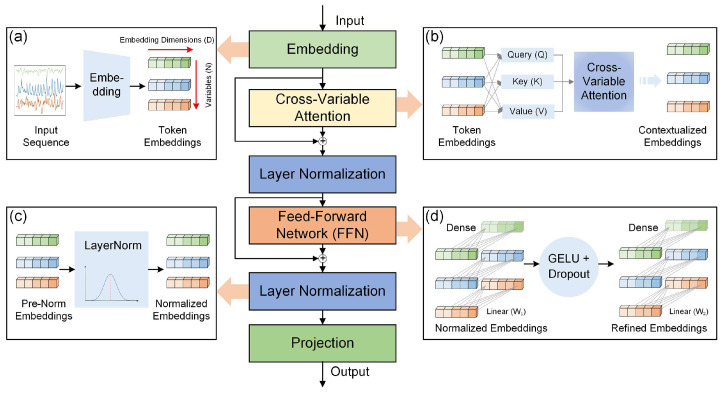
Structure of iTransformer. (**a**) Embedding; (**b**) Cross-variable attention; (**c**) Layer normalization; (**d**) Feed-forward network (FFN).

To address the variable mixing and misaligned temporal modeling issues in conventional Transformer-based multivariate time series predicting, we introduce the iTransformer architecture [43]. This method adopts an inverted modeling paradigm that reassigns the operational dimensions of self-attention and feed-forward networks, thereby enhancing the modeling of inter-variable dependencies and improving the temporal representation of individual variables (Figure 5).

The key idea of iTransformer is to treat each variable’s historical time series as an independent token and apply self-attention over the variable dimension to capture cross-variable dependencies, while using feed-forward networks along the temporal axis of each variable to extract long-term trends and short-term fluctuations. This design better preserves variable independence and enhances generalization capacity in predicting. The model input consists of multiple aligned time series variables, including GNSS displacement sequences in the X, Y, and Z directions, as well as multiscale cumulative precipitation features. These variables are jointly encoded to capture cross-domain interactions between kinematic deformation and hydrological forcing.

The procedure comprises the following steps:

Step 1: Embedding Each Variable’s Time Series

Given a multivariate time series with *N* variables and a lookback window of length *T*, iTransformer first processes each variable’s historical sequence independently. For each variable, the sequence of *T* time steps is mapped into a fixed-length embedding vector of dimension D. This embedding is typically produced by a multi-layer perceptron (MLP) and serves as the initial token representation for that variable. As a result, the input is transformed into a sequence of N token embeddings, each capturing the temporal characteristics of one variable over the lookback period.

Step 2: Modeling Cross-Variable Correlations (Self-Attention)

The token sequence $H^{\left(l\right)}\in R^{N\times D}$ is passed into a multi-head self-attention module applied over the variable dimension. For the l-th layer, the attention is computed as:

(3) $$Attention\left(Q,K,V\right)=Softmax(\frac{QK^{T}}{\sqrt{d_{k}}})V$$

where $Q,K,V\in R^{N\times d_{k}}$ represent the query, key, and value matrices, and $d_{k}$ is the attention projection dimension. This captures the dynamic dependency structure among variables.

Step 3: Layer Normalization with Residual Enhancement

The output of the attention module is processed via residual connection and layer normalization:

(4) $${\tilde{H}}^{\left(l\right)}=LayerNorm(H^{\left(l\right)}+Attention(H^{\left(l\right)}))$$

where ${\tilde{H}}^{\left(l\right)}\in R^{N\times D}$ is the normalized token output of layer *l*.

Step 4: Temporal Feature Extraction via Feed-Forward Network

A shared feed-forward network (FFN) is applied to each variable token independently, focusing on temporal pattern extraction such as trends, seasonality, and fluctuations:

(5) $$H^{\left(l+1\right)}=LayerNorm({\tilde{H}}^{\left(l\right)}+FFN({\tilde{H}}^{\left(l\right)}))$$

where *FFN* is typically a two-layer MLP.

Step 5: Sequence Prediction Generation

After several layers of processing, iTransformer obtains deep representations for each variable. These are then passed through a projection head to produce the final prediction values. The projection is applied independently per variable and supports arbitrary prediction lengths, yielding a multivariate output over the target prediction horizon.

The proposed PDM-iTransformer framework leverages the strengths of both structured decomposition and temporal abstraction. PDM offers interpretable multiscale analysis, while iTransformer ensures robust modeling of temporal dependencies. Together, they achieve enhanced predictive accuracy and generalization by aligning high-level trends with localized fluctuations. This integrated approach consistently outperforms standalone models, particularly in the context of landslide displacement predicting characterized by non-stationarity, irregular periodicity, and long-range dependencies [43].

### 3.3. Model Validation

To prepare for model training and evaluation, the dataset was split into training, validation, and testing subsets in a ratio of 70%, 15%, and 15%, respectively. The split was performed chronologically to preserve the temporal sequence and avoid information leakage between sets.

To comprehensively evaluate the performance of different prediction methods, three commonly used time-domain metrics were computed, namely the Coefficient of Determination (*R*^2^), Root Mean Square Error (*RMSE*), and Mean Absolute Error (*MAE*) [44,45]. These indicators provide a quantitative basis for comparing the predictive capabilities of each model under a unified evaluation framework. The metrics above are computed as follows:

(6) $$R^{2}=1-\frac{\sum_{i=1}^{n}{\left(y_{i}-{\hat{y}}_{i}\right)}^{2}}{\sum_{i=1}^{n}{\left(y_{i}-\bar{y}\right)}^{2}}$$

(7) $$RMSE=\sqrt{\frac{1}{n}\sum_{i=1}^{n}{\left(y_{i}-{\hat{y}}_{i}\right)}^{2}}$$

(8) $$MAE=\frac{1}{n}\sum_{i=1}^{n}\left|y_{i}-{\hat{y}}_{i}\right|$$

where *n* is the number of the testing samples, $y_{i}$ and ${\hat{y}}_{i}$ are the true values and predicted values, and $\bar{y}$ is the average of $y_{i}$.

In addition to time-domain assessments, normalized Power Spectral Density (PSD) analysis was introduced to evaluate model performance in the frequency domain [46,47]. The spectral energy distributions of predicted series were compared with those of the reference displacement data, enabling further insight into each algorithm in terms of trend preservation and high-frequency noise suppression.

## 4. Results and Discussion

### 4.1. Model Implementation and Prediction Accuracy

#### 4.1.1. Hyperparameter Settings

To ensure fair and consistent evaluation across models, we standardized the hyperparameter settings for all methods. For time-series neural models such as LSTM, CNN-LSTM, Transformer, and PDM-iTransformer, key hyperparameters include the sequence length, batch size, learning rate, and number of training epochs. The sequence length defines the historical time window used as input at each step and is essential for capturing temporal dependencies in displacement trends [48]. Batch size influences convergence speed and generalization performance, while the learning rate determines the step size during gradient descent. The number of epochs sets the maximum training iterations, and an early stopping strategy with a defined patience value was applied to avoid overfitting when the validation loss plateaued. In addition, for the Transformer and PDM-iTransformer models, we further specified structural and training-related parameters to ensure reproducibility and comparability. These include the number of encoder layers, embedding dimension, hidden size of the feed-forward network, activation function, dropout rate, weight decay, optimizer type, and learning rate scheduler. For the tree-based RF model, we adjusted parameters such as the number of estimators (n_estimators), maximum tree depth (max_depth), and the minimum number of samples required to split or form a leaf. These parameters control the ensemble’s capacity to fit complex patterns while mitigating overfitting risks. All hyperparameters were determined using a systematic search procedure to ensure reproducibility and objectivity. A grid search combined with five-fold cross-validation was employed to evaluate different hyperparameter configurations. Model performance was assessed using R^2^ and RMSE on the validation sets, and the configuration achieving the highest R^2^ and lowest RMSE was selected as the final setting. These optimized hyperparameters were subsequently fixed for all experiments to ensure consistent and comparable evaluation across different models. Detailed parameter configurations are summarized in Table 1. Before training, all deep learning models were applied with per-variable z-score standardization to each input feature. This step was performed separately for the three displacement directions (X, Y, Z) to eliminate differences in scale among variables and to prevent such discrepancies from biasing the attention weights.

#### 4.1.2. Time Series Prediction and Accuracy Verification

To comprehensively evaluate the performance of the proposed algorithm in landslide displacement prediction, statistical indicators between the predicted values and the measured truth were calculated for nine monitoring stations, as summarized in Table 2. To statistically validate the superiority of the proposed model, a paired two-tailed Student’s *t*-test was applied between the PDM-iTransformer and each baseline model (Transformer, CNN-LSTM, LSTM, RF) across the 27 station-direction combinations. The significance level was set to α = 0.05. *p*-values less than 0.05 were considered statistically significant.

According to the statistical evaluation of predictive performance across various models on GNSS-monitored landslide displacement data, the PDM-iTransformer consistently outperformed other models in all three key metrics: R^2^, RMSE, and MAE. It achieved an average R^2^ of 0.961, significantly surpassing Transformer (0.918), CNN-LSTM (0.827), LSTM (0.792), and RF (0.645), indicating superior capability in capturing nonlinear temporal dependencies. Moreover, it recorded the lowest average RMSE (0.770 mm) and MAE (0.614 mm), demonstrating enhanced accuracy and reduced prediction bias. PDM-iTransformer outperforms the standard Transformer because it treats the time series of each variable as an independent token for self-attention modeling, thereby avoiding interference caused by multivariate entanglement and improving the clarity and generalization ability in capturing cross-variable dependencies. Furthermore, its inverted architecture further enhances the model’s capacity to analyze both trend variations and local disturbances, making it more suitable for GNSS displacement sequences characterized by the coexistence of periodic noise and gradual trends. CNN-LSTM and LSTM models, based on gated recurrent units, exhibit better performance than RF due to their capacity to capture long-term temporal dependencies. However, they lack explicit global attention mechanisms and offer limited handling of multivariate interactions, making them less effective in complex sequential predicting compared to Transformer-based models. The RF model, having the lowest R^2^ and highest errors, shows limited capability in capturing nonlinear and temporal patterns. It performs better on static or tabular datasets but falls short in modeling spatial–temporal dynamics inherent in GNSS monitoring tasks.

Compared to traditional models such as CNN-LSTM, LSTM, and Random Forest, PDM-iTransformer improves R^2^ by 16.2–48.3%, while reducing RMSE and MAE by up to 1.33 mm and 1.08 mm, respectively. Even relative to the standard Transformer, it achieves a 4.6% higher R^2^ and lower error metrics. These results confirm that PDM-iTransformer offers the best combination of prediction accuracy and robustness, making it a highly promising approach for GNSS-based landslide displacement predicting in complex terrain settings. The paired *t*-test results showed that the PDM-iTransformer achieved significantly better performance than all baseline models across the three metrics (R^2^, RMSE, MAE), with all *p*-values < 0.001. These results confirm that the improvements are statistically significant and not due to random variability.

**Figure 6:**
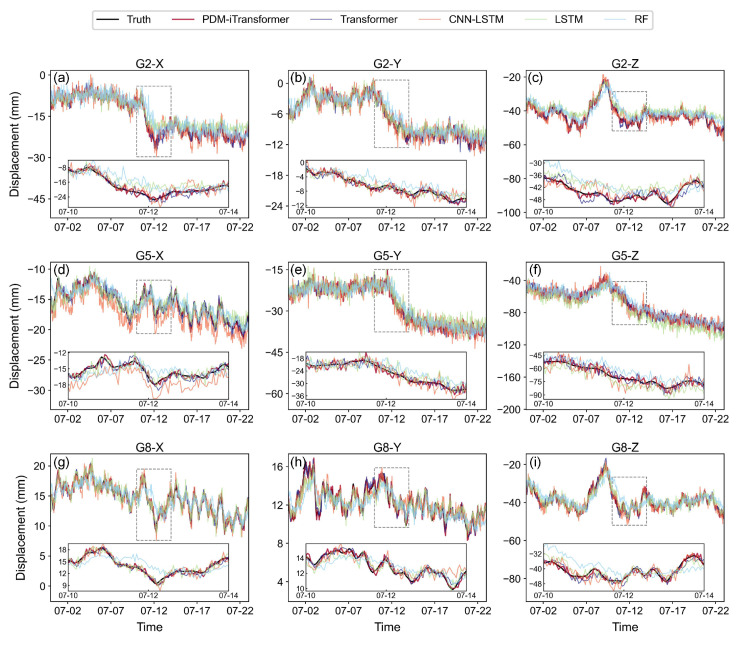
Comparison of time series prediction results at typical monitoring stations G2, G5, and G8. Each row corresponds to the results of stations G2, G5, and G8, respectively, while each column represents the X, Y, and Z displacement components. The subsequent figures follow the same sub-plot arrangement.

To provide a more intuitive comparison of time series prediction performance across different algorithms, three representative monitoring points (G2, G5, and G8) were selected for detailed analysis, as shown in Figure 6. These points, located in the central area of the landslide body, offer good continuity and representativeness, effectively reflecting displacement characteristics in the X, Y, and Z directions and allowing comparative evaluation of model performance.

As shown in Figure 6, the proposed PDM-iTransformer generally achieves the closest alignment with the ground truth across most stations and directions. In examples like G8-Y and G5-Y, its predicted curves closely follow the measured data, demonstrating strong capability in capturing both trend and short-term fluctuations. Even in the presence of abrupt changes or high-frequency variations, the model maintains smooth and continuous responses, reflecting its effectiveness in modeling complex nonlinear displacement behavior. This benefit comes from its inverted structure and fine-grained temporal modeling with attention mechanisms. Importantly, localized error behaviors can also be observed in segments of abrupt displacement changes, such as around July 11 in G2-Z and G5-Z. In these periods, PDM-iTransformer maintains continuity, while other models such as CNN-LSTM and RF show delayed or oscillatory responses. This highlights PDM-iTransformer’s superior ability in timely and accurate response to sudden slope changes.

Furthermore, the models’ capacity to capture overall deformation trends and filter local noise is also evident. For example, in G5-Y, the slow downward displacement trend is well captured by PDM-iTransformer and Transformer, whereas LSTM and RF tend to deviate with overshooting or instability. In relatively stable segments such as G8-X, PDM-iTransformer preserves the underlying signal while suppressing small high-frequency disturbances, whereas RF and CNN-LSTM amplify short-term noise.

The standard Transformer also performs well in several directions, notably in G8-X, benefiting from global attention and sequence encoding. However, it exhibits minor overfitting and noise amplification in cases like G2-Y and G2-Z, which may result from entangled multivariate representations. The LSTM model, while maintaining relative stability, underperforms in directions such as G2-X and G8-Y, where its responses tend to lag and underestimate displacement changes—due to its limited memory horizon and lack of explicit attention. The RF model shows significant issues in several directions including G2-Z, G5-X, G5-Z, and G8-Z, with oscillatory predictions, delays, and misalignment from the actual values. Its tree-based structure is inherently weak in temporal dependency modeling. CNN-LSTM shows moderate performance, with reasonable accuracy in G8-Y, though it lacks responsiveness in capturing sharp changes such as in G2-Z. Its fixed convolution window and sequential gating offer partial pattern capture, but struggle with irregular dynamics.

On the whole, the results underscore the superior accuracy, robustness, and sensitivity of the PDM-iTransformer in modeling complex and non-stationary landslide displacement processes.

### 4.2. Frequency-Domain Characteristics Analysis

**Figure 7:**
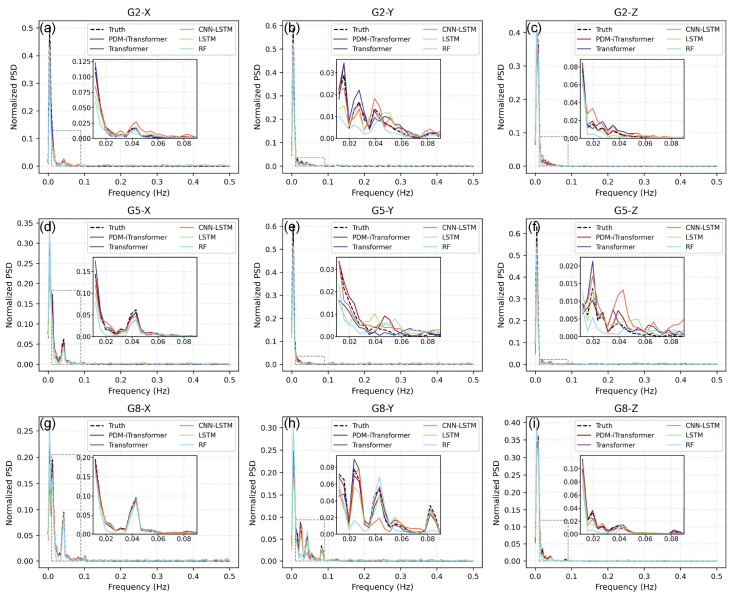
Comparison of normalized power spectral density of different models.

To further investigate the ability of each model to capture the frequency-domain characteristics of landslide displacement signals, this study introduces PSD analysis to evaluate the consistency between the predicted sequences and the ground truth in the spectral domain, as shown in Figure 7. This method reveals how well each model captures the long-term trends (low-frequency components) and short-term fluctuations (high-frequency components) of landslide deformation, thereby reflecting the model’s stability and smoothness.

In Figure 7, the proposed PDM-iTransformer model exhibits a spectral distribution that closely matches the ground truth in many monitoring stations and displacement directions. In the low-frequency range from 0.01 to 0.05 Hz, which reflects long-term landslide deformation, the model effectively reconstructs the dominant spectral energy, demonstrating strong capability in preserving slow-varying trends in displacement. This stems from its attention-based architecture, which enables fine-grained learning of temporal dynamics. Beyond general alignment, further spectral phenomena can be observed. First, models like RF and CNN-LSTM often show a noticeable rightward shift in the spectral peaks compared to the ground truth, particularly in directions such as G2-Z and G5-Y. This indicates a tendency to amplify higher-frequency content, potentially reducing their ability to capture long-term trends. In contrast, PDM-iTransformer preserves the dominant peak frequency more accurately. Second, the spectral curves of RF, CNN-LSTM, and even Transformer tend to exhibit broader frequency bands and slower energy decay after the peak, suggesting a greater spread of energy into mid- and high-frequency ranges. This reflects more high-frequency noise and less temporal smoothness in the predictions. Conversely, PDM-iTransformer’s spectral energy is more concentrated, indicating better regularization and smoother outputs.

In comparison, the RF model shows a consistent shift of spectral energy toward the high-frequency region in several directions, indicating that its predictions are more affected by noise and lack temporal smoothness due to its non-sequential tree-based structure. The CNN-LSTM and LSTM models provide partial improvements over RF in the low and mid-frequency bands. However, in certain subplots, their predictions still retain non-negligible high-frequency energy, suggesting that their sequential memory may be insufficient for fully suppressing short-term fluctuations. The standard Transformer performs relatively well in capturing low-frequency components, especially in directions such as G8-Y and G5-Y. However, in certain directions like G2-Y, it exhibits energy concentration in the mid-frequency range, which may contribute to oscillatory components in the predicted signals. Significantly, in the G5-Z direction, the CNN-LSTM model displays a noticeably elevated normalized spectral density in the mid-to-high frequency range, implying a higher degree of noise or instability.

The magnified insets focusing on the 0.01 to 0.09 Hz frequency band further illustrate the superior trend-preserving performance of the PDM-iTransformer. Its spectral curves closely resemble the ground truth in both magnitude and shape, particularly in G2-Z, G5-X, and G8-Z, where other models show visible deviations. These frequency-domain results provide strong evidence for the PDM-iTransformer’s effectiveness in preserving long-term trends, mitigating high-frequency noise, and improving the spectral consistency of landslide displacement predictions.

**Figure 8:**
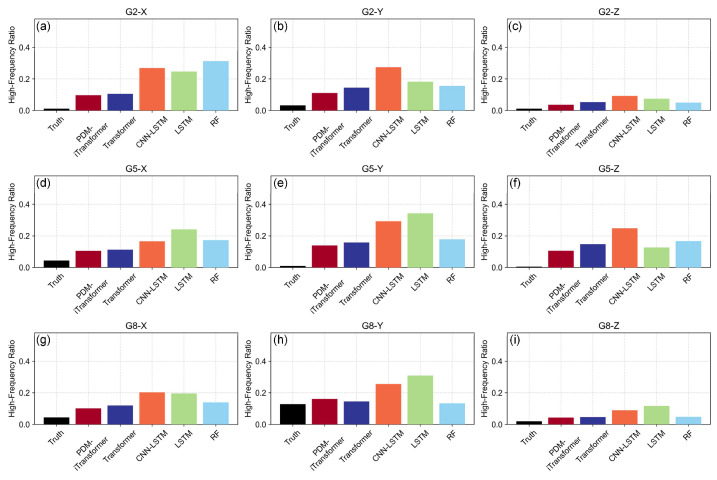
Comparison of high-frequency power ratios (>0.05 Hz) of different models.

Moreover, the high-frequency power ratio, which refers to the proportion of spectral energy contained in frequency components exceeding 0.05 Hz, is calculated to quantify the extent of high-frequency fluctuations in the predicted sequences at each station (Figure 8). Unlike conventional error metrics in the time domain, this indicator emphasizes the distribution of signal energy across the frequency spectrum, offering valuable insights into potential issues such as noise amplification, model overfitting, and distortion of spectral characteristics in the predicted results.

As shown in Figure 8, the PDM-iTransformer model exhibits the lowest or near-lowest high-frequency energy ratios across most directions and monitoring stations, indicating strong capability in preserving the smoothness of long-term deformation trends. This spectral advantage reflects its architectural design that suppresses short-term noise while maintaining trend continuity. In contrast, the RF model often displays relatively higher high-frequency components, particularly in directions such as G2-X, G5-Z, and G8-X, suggesting its predictions are more susceptible to temporal noise. The LSTM and CNN-LSTM models perform slightly better than RF in some directions such as G2-Z and G8-Y, but still exhibit elevated high-frequency ratios in cases like G2-X, G5-X, and G5-Z. This reflects their limited ability to suppress short-term disturbances due to lack of global attention and temporal filtering mechanisms. The standard Transformer shows moderate but non-negligible high-frequency power ratios in most directions. While generally smoother than LSTM-based models, it still retains some residual spectral energy in mid-to-high frequency bands. Overall, the PDM-iTransformer demonstrates superior spectral control, effectively reducing non-trend high-frequency fluctuations and producing cleaner prediction sequences that are better suited for subsequent trend evaluation and early warning applications.

### 4.3. Prediction Uncertainty and Temporal Sensitivity Analysis

**Figure 9:**
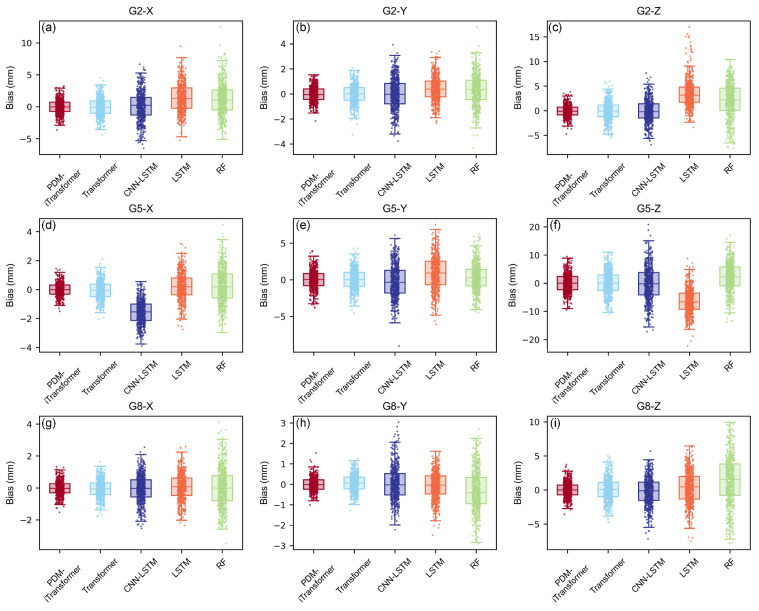
Boxplot of absolute bias of different models.

To comprehensively evaluate the prediction error characteristics of different models, boxplots of absolute deviations between predicted and true values were generated across three typical stations. The median errors, interquartile ranges, and distributions of outliers for each model are shown in Figure 9.

From Figure 9, the results show that the proposed PDM-iTransformer achieves the lowest or near-lowest median residuals across most directions, with consistently narrow interquartile ranges, indicating better stability and accuracy in its predictions. In contrast, the RF model exhibits substantial variability, particularly in directions such as G2-Z and G5-Z, where it shows many extreme outliers and wider dispersion. Similarly, CNN-LSTM displays pronounced outliers in some directions (e.g., G2-Z, G5-Z), suggesting limited robustness in complex displacement scenarios. The LSTM and CNN-LSTM models perform similarly to RF in terms of residual dispersion, although they present slightly fewer extreme bias values in some cases such as G8-Y and G2-Y. The Transformer model performs stably in many directions (e.g., G2-X, G5-X, G8-X), with moderate spread and balanced error distribution, though occasional deviations still exist.

These findings further confirm the robustness and predictive reliability of the PDM-iTransformer for GNSS-based landslide displacement prediction, particularly in controlling bias, reducing variability, and suppressing prediction outliers.

**Figure 10:**
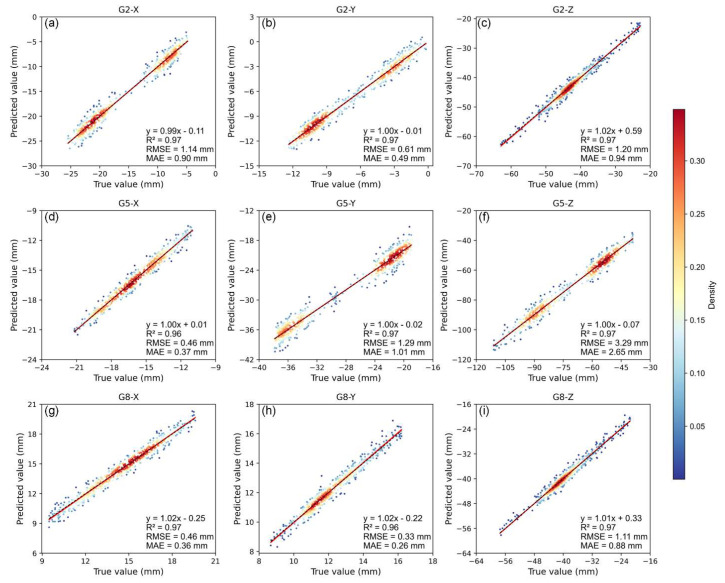
Scatter plots of PDM-iTransformer predictions.

To evaluate the predictive stability and bias dispersion of the proposed method, scatter plots of predicted values versus ground truth are presented for the representative monitoring points G2, G5, and G8 across the X, Y, and Z directions. As shown in Figure 10, the PDM-iTransformer model exhibits strong linear correlations in all subplots, with R^2^ values exceeding 0.96. The scatter points are generally well aligned with the 1:1 reference line, indicating high consistency and low average bias. Notably, in directions such as G5-X, G8-X, and G8-Y, the scatter points are tightly clustered along the diagonal, and the fitted regression lines closely follow the identity line, demonstrating the model’s ability to accurately track displacement magnitudes while maintaining temporal coherence. However, G5-Z and G8-Z show more dispersed distributions, where several points deviate from the ideal trend. These deviations are consistent with their higher RMSE and MAE values and may be caused by non-stationary noise or localized discontinuities in deformation patterns. In general, the results confirm that the PDM-iTransformer provides stable and accurate predictions, with minimal bias and strong agreement between predicted and observed displacement sequences in most scenarios.

**Figure 11:**
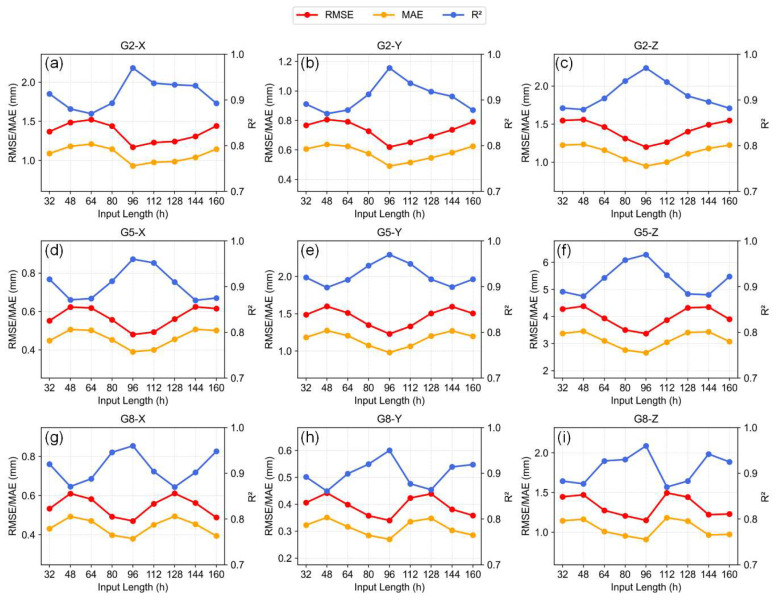
Prediction performance of PDM-iTransformer under different input sequence lengths.

In order to assess the prediction stability and temporal sensitivity of the model under varying input lengths, Figure 11 illustrates the performance of the PDM-iTransformer across representative stations and three displacement directions. During these experiments, all other hyperparameters (e.g., learning rate, number of layers, batch size, embedding dimension, dropout, optimizer, and learning rate scheduler) were kept constant and identical to those listed in Table 1, ensuring that any performance variation is solely attributed to the change in sequence length. To account for the stochastic nature of model training, each experiment was repeated five times using different random seeds, and the results are reported as mean ± standard deviation (Appendix A Table A1).

In general, the 96 h (i.e., 4-day) input length consistently yields the best prediction accuracy across all scenarios, achieving the highest R^2^ and lowest RMSE and MAE values. This suggests that the model effectively balances the capture of long-term displacement trends with suppression of excessive noise. Several local optima are also observed at input lengths such as 64 and 128 h, indicating the model’s robustness to moderate variations in temporal scale. Additionally, the error fluctuation characteristics vary across directions—for instance, directions like G2-Z and G5-Z exhibit smoother error trends, implying higher predictability, while G2-X and G5-Z show larger performance fluctuations, potentially influenced by noise levels or terrain complexity. Based on these findings, the 96 h sequence is selected as the optimal hyperparameter, reflecting the temporal characteristics of landslide evolution while ensuring model generalization and training stability.

## 5. Discussion

This study introduces a novel PDM-iTransformer prediction framework designed to decompose displacement sequences into multi-resolution components and independently model each variable through self-attention. This approach enables the effective characterization of both long-term deformation trends and localized disturbances. In comparison with other baseline models, the superior performance of the PDM-iTransformer can be attributed to its distinctive architectural design. By treating each displacement direction as an independent token, the framework minimizes interference arising from multivariate interactions, thereby improving clarity and enhancing the ability to capture cross-variable dependencies. The PDM module allows the model to represent both gradual, slow-varying trends and rapidly changing components, while the inverted Transformer structure strengthens its capacity to model hierarchical temporal dependencies. These features make the PDM-iTransformer particularly well suited for GNSS monitoring data, which are often characterized by non-stationary behavior, noise contamination, and the simultaneous presence of periodic and abrupt deformation patterns. From an application perspective, these capabilities are highly valuable for real-time monitoring and decision-making because they provide more reliable early warning signals and support proactive strategies for disaster risk reduction.

Although the results of this study are promising, there remain opportunities for refinement and further development to broaden the scope and applicability of the proposed framework. First, the current validation was based on data from a single landslide site in Chifeng, China, which was selected as a representative case. While this provides valuable insights into the model’s performance, additional testing on other stations with different geological, climatic, or deformation conditions would help to further demonstrate its adaptability. Variations in factors such as soil composition, slope geometry, or rainfall patterns may lead to differences in model behavior, and future studies will explore these aspects to improve generalizability. Second, the dataset used in this study covered a 160-day monitoring period that primarily reflects the rainy season and associated peak landslide activity. This timeframe successfully captured key deformation events, although longer-term observations would offer a more comprehensive view of seasonal variations and gradual creep processes. Extending the monitoring period to encompass a full annual cycle or multi-year observations could provide additional data for model calibration and validation, further strengthening its reliability. In addition, future work will consider integrating supplementary environmental variables, such as soil moisture, pore water pressure, temperature, and seismic activity, to enhance predictive performance and support broader applications in complex early warning scenarios.

## 6. Conclusions

This study presents a hybrid framework, PDM-iTransformer, for GNSS-based landslide displacement prediction under nonstationary and multiscale temporal dynamics. By integrating structured decomposition with attention-based modeling, the framework offers an interpretable and robust approach that effectively captures both kinematic and hydrological influences on slope movement. The main contributions and findings are summarized as follows:

(1) PDM enables multiscale feature extraction and interpretable decomposition, effectively isolating trend and seasonal components of GNSS displacement sequences. This enhances the model’s ability to represent complex temporal variations, contributing to improved prediction robustness.
(2) The iTransformer architecture captures cross-variable interactions between displacement and precipitation features, leveraging variable-wise attention and temporal abstraction to model nonlinear dependencies. This facilitates joint modeling of kinematic deformation and precipitation-induced triggers.
(3) The combined framework achieves consistent performance gains over existing models, with R^2^ improved by 16.2–48.3%, and RMSE/MAE reduced by up to 1.33 mm and 1.08 mm, respectively. These improvements reflect the synergy between multiscale decomposition and attention-based modeling in capturing long-term trends and short-term fluctuations.

In summary, the PDM-iTransformer framework provides a unified and effective solution for landslide displacement predicting. With further integration of heterogeneous geohazard sensing data, the proposed method could be extended beyond landslide prediction to other scenarios, such as debris flow warning and slope stability monitoring under complex and dynamic environmental conditions.

## Figures and Tables

**Table 1:** Hyperparameter settings of different methods.

Method	Hyperparameter	Explanation
PDM- iTransformer	sequence length = 96, batch size = 24, attention heads = 8, learning rate = 1 × 10^−4^, epoch = 150, patience = 15, layers = 4, D = 128, FFN_hidden = 256, activation = GELU, dropout = 0.2, weight_decay = 1 × 10^−4^, optimizer = AdamW, lr_scheduler = Cosine decay	sequence length: Number of past time steps used as input. batch size: Number of samples per training batch. attention heads: Number of attention heads in multi-head attention. learning rate: Step size for optimizer updates. epoch: Maximum number of training iterations. patience: Number of epochs with no improvement before early stopping. cnn_filters: Number of filters in CNN layers. lstm_units: Number of hidden units in LSTM layers. dropout: Dropout rate for regularization. layers: Number of encoder layers in the Transformer or iTransformer. D: Embedding dimension of input features for each attention head. FFN_hidden: Number of hidden units in the feed-forward network. activation: Type of activation function used in the network. dropout: Dropout rate for regularization to prevent overfitting. weight_decay: L2 regularization parameter for controlling model complexity. optimizer: Optimization algorithm used for gradient updates. lr_scheduler: Learning rate adjustment strategy during training.
Transformer	sequence length = 96, batch size = 24, attention heads = 8, learning rate = 1 × 10^−4^, epoch = 150, patience = 15, layers = 4, D = 128, FFN_hidden = 256, activation = GELU, dropout = 0.2, weight_decay = 1 × 10^−4^, optimizer = AdamW, lr_scheduler = Cosine decay	
CNN-LSTM	sequence length = 96, batch size = 24, cnn_filters = 64, lstm_units = 128, dropout = 0.2, learning rate = 1 × 10^−4^, epoch = 150, patience = 15	
LSTM	sequence length = 96, batch size = 24, lstm_units = 128, dropout = 0.2, learning rate = 1 × 10^−4^, epoch= 150, patience = 15	
RF	n_estimators = 500, max_depth = 20, min_samples_split = 4, min_samples_leaf = 2	n_estimators: Number of decision trees. max_depth: Maximum depth of each tree. min_samples_split: Minimum number of samples required to split an internal node. min_samples_leaf: Minimum number of samples required to be at a leaf node.

**Table 2:** Statistical indicators of different methods at nine monitoring stations.

Station	Axis	R^2^					RMSE (mm)					MAE (mm)				
		PDM- iTrans- former	Trans- former	CNN- LSTM	LSTM	RF	PDM- iTrans former	Trans- former	CNN- LSTM	LSTM	RF	PDM- iTrans- former	Trans- former	CNN- LSTM	LSTM	RF
G1	X	0.96	0.93	0.82	0.81	0.5	0.39	0.5	0.8	0.82	1.32	0.31	0.4	0.64	0.65	1.07
	Y	0.95	0.9	0.78	0.84	0.44	0.28	0.41	0.6	0.52	0.96	0.23	0.32	0.47	0.4	0.75
	Z	0.97	0.92	0.86	0.86	0.66	1.27	1.98	2.67	2.67	4.16	1.01	1.56	2.11	2.12	3.34
G2	X	0.97	0.96	0.89	0.85	0.84	1.14	1.38	2.19	2.59	2.71	0.9	1.11	1.73	2.04	2.11
	Y	0.97	0.96	0.89	0.92	0.89	0.61	0.77	1.22	1.06	1.25	0.49	0.61	0.98	0.84	1
	Z	0.97	0.91	0.88	0.53	0.62	1.2	1.91	2.26	4.49	4	0.94	1.51	1.78	3.62	3.33
G3	X	0.96	0.94	0.85	0.88	0.61	0.39	0.5	0.8	0.71	1.29	0.31	0.39	0.63	0.58	1.04
	Y	0.95	0.88	0.8	0.81	0.4	0.25	0.39	0.5	0.49	0.87	0.2	0.31	0.4	0.39	0.68
	Z	0.97	0.93	0.89	0.67	0.69	1.2	1.68	2.21	3.78	3.65	0.97	1.33	1.75	3.02	2.97
G4	X	0.96	0.93	0.87	0.88	0.66	0.39	0.5	0.7	0.66	1.11	0.31	0.4	0.56	0.54	0.92
	Y	0.94	0.79	0.69	0.64	0.51	0.31	0.61	0.75	0.8	0.94	0.24	0.5	0.57	0.62	0.76
	Z	0.96	0.92	0.89	0.83	0.67	1.21	1.78	2.11	2.63	3.66	0.97	1.41	1.69	2.11	3.02
G5	X	0.96	0.93	0.45	0.84	0.73	0.46	0.64	1.76	0.95	1.23	0.37	0.51	1.58	0.75	0.99
	Y	0.97	0.96	0.9	0.87	0.93	1.29	1.39	2.23	2.5	1.9	1.01	1.1	1.81	2.02	1.46
	Z	0.97	0.95	0.9	0.83	0.92	3.29	4.06	5.9	7.84	5.47	2.65	3.26	4.7	6.81	4.41
G6	X	0.96	0.94	0.88	0.84	0.67	0.36	0.45	0.66	0.76	1.09	0.29	0.36	0.53	0.6	0.87
	Y	0.96	0.91	0.7	0.67	0.64	0.3	0.46	0.82	0.86	0.9	0.24	0.36	0.62	0.66	0.72
	Z	0.96	0.92	0.89	0.49	0.69	1.09	1.52	1.85	3.94	3.08	0.85	1.22	1.47	3.04	2.53
G7	X	0.96	0.94	0.86	0.83	0.55	0.34	0.39	0.62	0.66	1.09	0.27	0.31	0.49	0.53	0.89
	Y	0.95	0.81	0.72	0.63	0.43	0.26	0.51	0.62	0.71	0.88	0.21	0.4	0.49	0.54	0.69
	Z	0.96	0.93	0.88	0.85	0.67	1.19	1.63	2.18	2.49	3.63	0.96	1.3	1.74	1.99	2.89
G8	X	0.96	0.95	0.88	0.88	0.75	0.46	0.56	0.83	0.84	1.21	0.36	0.45	0.65	0.67	0.96
	Y	0.95	0.92	0.72	0.81	0.54	0.33	0.43	0.8	0.67	1.03	0.26	0.34	0.62	0.54	0.85
	Z	0.96	0.92	0.88	0.83	0.57	1.11	1.62	2.03	2.43	3.81	0.88	1.28	1.62	1.97	3.08
G9	X	0.96	0.93	0.87	0.84	0.64	0.35	0.49	0.65	0.73	1.09	0.28	0.39	0.5	0.58	0.88
	Y	0.96	0.87	0.82	0.81	0.55	0.26	0.47	0.55	0.57	0.88	0.21	0.37	0.44	0.46	0.7
	Z	0.97	0.93	0.88	0.84	0.64	1.07	1.47	2.02	2.28	3.43	0.86	1.17	1.62	1.82	2.78

## Data Availability

The data supporting the findings of this study are available from the corresponding author upon reasonable request.

## Appendix A

**Table A1:** Statistical metrics of PDM-iTransformer under different input sequence lengths.

Station	Axis	t	R^2^	RMSE (mm)	MAE (mm)
G2	X	32	0.913 ± 0.010	1.369 ± 0.010	1.089 ± 0.009
		48	0.880 ± 0.014	1.485 ± 0.009	1.181 ± 0.008
		64	0.870 ± 0.012	1.521 ± 0.007	1.209 ± 0.007
		80	0.893 ± 0.012	1.440 ± 0.009	1.144 ± 0.008
		96	0.970 ± 0.009	1.170 ± 0.009	0.930 ± 0.008
		112	0.937 ± 0.009	1.228 ± 0.011	0.977 ± 0.010
		128	0.933 ± 0.008	1.243 ± 0.010	0.986 ± 0.009
		144	0.931 ± 0.013	1.306 ± 0.011	1.039 ± 0.010
		160	0.893 ± 0.012	1.441 ± 0.009	1.145 ± 0.008
	Y	32	0.891 ± 0.012	0.767 ± 0.008	0.607 ± 0.007
		48	0.870 ± 0.008	0.806 ± 0.011	0.637 ± 0.010
		64	0.878 ± 0.014	0.792 ± 0.011	0.625 ± 0.010
		80	0.912 ± 0.013	0.727 ± 0.010	0.575 ± 0.009
		96	0.970 ± 0.009	0.620 ± 0.011	0.490 ± 0.010
		112	0.937 ± 0.009	0.651 ± 0.009	0.515 ± 0.008
		128	0.918 ± 0.009	0.692 ± 0.010	0.547 ± 0.009
		144	0.908 ± 0.010	0.736 ± 0.009	0.581 ± 0.008
		160	0.878 ± 0.011	0.790 ± 0.007	0.625 ± 0.007
	Z	32	0.882 ± 0.011	1.548 ± 0.008	1.226 ± 0.007
		48	0.879 ± 0.010	1.560 ± 0.007	1.235 ± 0.007
		64	0.903 ± 0.012	1.464 ± 0.010	1.159 ± 0.009
		80	0.942 ± 0.009	1.312 ± 0.009	1.039 ± 0.008
		96	0.970 ± 0.010	1.200 ± 0.010	0.950 ± 0.009
		112	0.939 ± 0.010	1.263 ± 0.012	1.000 ± 0.011
		128	0.909 ± 0.011	1.403 ± 0.008	1.111 ± 0.007
		144	0.896 ± 0.013	1.493 ± 0.009	1.182 ± 0.008
		160	0.882 ± 0.009	1.549 ± 0.011	1.226 ± 0.010
G5	X	32	0.916 ± 0.011	0.552 ± 0.008	0.448 ± 0.007
		48	0.871 ± 0.012	0.623 ± 0.007	0.506 ± 0.007
		64	0.874 ± 0.008	0.618 ± 0.008	0.502 ± 0.007
		80	0.912 ± 0.012	0.556 ± 0.008	0.452 ± 0.007
		96	0.960 ± 0.009	0.480 ± 0.012	0.390 ± 0.011
		112	0.952 ± 0.008	0.493 ± 0.011	0.400 ± 0.010
		128	0.910 ± 0.014	0.560 ± 0.010	0.455 ± 0.009
		144	0.870 ± 0.014	0.624 ± 0.011	0.507 ± 0.010
		160	0.875 ± 0.013	0.615 ± 0.011	0.501 ± 0.010
	Y	32	0.920 ± 0.010	1.486 ± 0.008	1.183 ± 0.007
		48	0.898 ± 0.009	1.599 ± 0.011	1.274 ± 0.010
		64	0.915 ± 0.012	1.512 ± 0.010	1.205 ± 0.009
		80	0.946 ± 0.011	1.350 ± 0.011	1.075 ± 0.010
		96	0.970 ± 0.009	1.230 ± 0.011	0.980 ± 0.010
		112	0.950 ± 0.011	1.331 ± 0.009	1.064 ± 0.008
		128	0.916 ± 0.008	1.505 ± 0.008	1.202 ± 0.007
		144	0.899 ± 0.013	1.596 ± 0.008	1.270 ± 0.007
		160	0.916 ± 0.010	1.505 ± 0.009	1.198 ± 0.008
	Z	32	0.889 ± 0.012	4.276 ± 0.011	3.376 ± 0.010
		48	0.879 ± 0.010	4.381 ± 0.011	3.458 ± 0.010
		64	0.919 ± 0.011	3.931 ± 0.007	3.102 ± 0.007
		80	0.958 ± 0.011	3.502 ± 0.010	2.763 ± 0.009
		96	0.970 ± 0.009	3.370 ± 0.009	2.660 ± 0.008
		112	0.925 ± 0.014	3.864 ± 0.008	3.049 ± 0.007
		128	0.884 ± 0.013	4.320 ± 0.008	3.410 ± 0.007
		144	0.882 ± 0.014	4.349 ± 0.009	3.434 ± 0.008
		160	0.922 ± 0.013	3.899 ± 0.012	3.078 ± 0.011
G8	X	32	0.920 ± 0.012	0.533 ± 0.009	0.431 ± 0.008
		48	0.871 ± 0.014	0.610 ± 0.010	0.493 ± 0.009
		64	0.888 ± 0.009	0.582 ± 0.011	0.471 ± 0.010
		80	0.946 ± 0.009	0.492 ± 0.009	0.398 ± 0.008
		96	0.960 ± 0.008	0.470 ± 0.012	0.380 ± 0.011
		112	0.904 ± 0.010	0.558 ± 0.012	0.451 ± 0.011
		128	0.870 ± 0.010	0.611 ± 0.008	0.494 ± 0.007
		144	0.902 ± 0.010	0.562 ± 0.009	0.454 ± 0.008
		160	0.948 ± 0.013	0.488 ± 0.009	0.394 ± 0.008
	Y	32	0.892 ± 0.010	0.406 ± 0.008	0.323 ± 0.007
		48	0.861 ± 0.010	0.442 ± 0.007	0.351 ± 0.007
		64	0.899 ± 0.011	0.399 ± 0.010	0.317 ± 0.009
		80	0.920 ± 0.009	0.358 ± 0.010	0.285 ± 0.009
		96	0.950 ± 0.013	0.340 ± 0.007	0.270 ± 0.007
		112	0.877 ± 0.008	0.423 ± 0.008	0.335 ± 0.007
		128	0.864 ± 0.014	0.439 ± 0.012	0.348 ± 0.011
		144	0.914 ± 0.013	0.381 ± 0.008	0.303 ± 0.007
		160	0.919 ± 0.009	0.359 ± 0.008	0.286 ± 0.007
	Z	32	0.883 ± 0.008	1.447 ± 0.009	1.145 ± 0.008
		48	0.877 ± 0.013	1.470 ± 0.012	1.163 ± 0.011
		64	0.927 ± 0.012	1.275 ± 0.008	1.009 ± 0.007
		80	0.930 ± 0.012	1.207 ± 0.010	0.955 ± 0.009
		96	0.960 ± 0.013	1.150 ± 0.011	0.910 ± 0.010
		112	0.870 ± 0.008	1.495 ± 0.008	1.183 ± 0.007
		128	0.883 ± 0.010	1.443 ± 0.011	1.142 ± 0.010
		144	0.942 ± 0.009	1.221 ± 0.009	0.966 ± 0.008
		160	0.925 ± 0.013	1.230 ± 0.010	0.973 ± 0.009
